# Effect of Silver Promoter on the H_2_ Gasochromic Recovery Behavior of Pt-Decorated WO_3_ Nanowires

**DOI:** 10.3390/ijms27020833

**Published:** 2026-01-14

**Authors:** Dandan Liu, Ziheng Geng, Aiyan Han, Rongjiao Che, Ping Yu, Huan Liu, Yunqi Liu

**Affiliations:** 1 State Key Laboratory of Heavy Oil Processing, College of New Energy, College of Chemistry and Chemical Engineering, China University of Petroleum (East China), Qingdao 266580, China; liudandan@upc.edu.cn (D.L.); 17854258270@163.com (Z.G.); cherongjiao2023@163.com (R.C.); 2 State Key Laboratory of Chemical Safety, Sinopec Research Institute of Safety Engineering Co., Ltd., Qingdao 266000, China; yup.qday@sinopec.com

**Keywords:** tungsten oxide, hydrogen gasochromic, recovery, silver, oxygen

## Abstract

The hydrogen gasochromic phenomenon offers a new strategy for real-time sensing technologies for hydrogen leakage to ensure hydrogen safety. However, the limited recovery kinetics impede the cycling and further practical applications. Herein, we designed a series of PtAg-decorated WO_3_ nanowires via the chemical reduction deposition method, which could exhibit obvious and reversible color changes for H_2_ detection. With the assistance of Ag, the oxygen adsorption and dissociation were accelerated; then, the sample could exhibit a constant rapid recovery rate. The crystalline Pt-Ag/WO_3_ nanowires could attain a 50% recovery degree within 52 s, and the recovery time of the Pt-Ag/WO_3_ sample was reduced to one fifth that of Pt/WO_3_. This study provides a fundamental solution to the challenge of slow recovery kinetics in H_2_ gasochromic crystalline materials.

## 1. Introduction

With the rapid development of hydrogen energy, advancing safety assurance technologies is crucial to support the widespread application of hydrogen [1,2]. The chromogenic phenomenon provides a visible method of detecting hydrogen, offering a convenient, sensitive, and cost-effective approach for monitoring hydrogen leakage, particularly at low concentrations [3,4]. WO_3_ is a representative gasochromic material for hydrogen detection, which can change color from light yellow to dark blue upon exposure to hydrogen [5,6,7]. However, this process should be facilitated by noble metals such as Pt and Pd, which could promote hydrogen dissociation, enhance sensitivity, modulate selectivity, and reduce the operating temperature for hydrogenochromic phenomena [4,5]. Typically, the hydrogen gasochromic behavior of WO_3_ is revisable. When the material in a colored state is re-exposed to oxygen or air, it can revert to its original color. The current research has mainly focused on enhancing sensitivity to hydrogen; however, achieving the recovery of colored material in mild conditions is still a challenge for the recycling of hydrogen gasochromic materials. Mild recovery conditions will significantly facilitate the reuse of visual hydrogen-sensitive devices.

The Jang group designed a H_2_ sensor using Pd-decorated amorphous WO_3_ nanorods with rapid recovery properties. They proposed that the porous amorphous nanostructure with a high surface-to-volume ratio accounted for the rapid recovery of sensors [8]. They also found that the dissociated H^+^ in the interstitial position of WO_3_ can easily diffuse in and out of the amorphous WO_3_, thus facilitating the recovery [9]. The Seo group also reported that a WO_3_ film with an amorphous structure could improve the adsorption and diffusion of hydrogen inside bulk WO_3_, but this film presented degraded properties during the continuous switching between H_2_ and air [10]. The crystalline WO_3_ could exhibit a rapid response at a low concentration of H_2_, but the elongated recovery process is unfavorable for the investigation of recycling stability and recovery mechanisms in the oxygenous atmosphere [11,12,13]. This is mainly because the crystalline WO_3_ could exist as a stable H_x_WO_3_ structure, thus suffering from incomplete recovery or slow recovery kinetics [13,14]. However, the present report mainly concentrates on the spillover of hydrogen during the recovery process, ignoring the oxygen activation process during recovery. According to the local water molecule model, enhancing the generation rate of activated oxygen on the material surface is identified as the critical factor controlling the recovery kinetics [15]. In addition, WO_3_ only favors the generation of hydroxyl radical thermodynamically due to the weakness of the conduction-band potential [16], which also limits the kinetics of the recovery process. To overcome this bottleneck, introducing abundant oxygen active species to manipulate the generation and spillover of reactive oxygen species on WO_3_ is important.

Although Pt could realize efficient hydrogen dissociation under mild conditions, the ability for oxygen activation could not satisfy the requirements of the recovery process [13,17]. The effective recovery of colored materials under mild conditions can be realized by modulating the chemical and physical structures of catalysts [7,8,17,18,19]. Among these modulation strategies, bimetallic active sites could provide tunable electronic structures and synergistic effects compared with monometal active sites, facilitating the creation of favorable oxygen-active environments and efficient catalytic oxidation systems. Sun-Woo Choi et al. [20] demonstrated that bimetallic Pd/Pt-functionalized oxide could significantly enhance the sensitivity to NO_2_ and reduce the response and recovery times, which is attributed to the unique structure and the synergistic effect of Pd and Pt. The different metal components could influence the catalytic oxidation (oxygen spillover effect) and electronic sensitization (Fermi energy control) of metal active sites, thus effectively affecting the recovery behavior [21,22,23]. Therefore, the preparation of WO_3_ decorated with Pt and metal promoters is an effective way to achieve high response and rapid recovery kinetics during the reversible hydrogen gasochromic process.

In this study, various second metal promoters were introduced to assist the recovery process of Pt-decorated WO_3_ nanowire. Based on the gasochromic performance, silver was identified as the optimal secondary metal promoter, which effectively improved the recovery stage. To elucidate the promotional role of Ag, the PtAg metallic active sites with tailored microstructures were synthesized, and the improvement mechanism of Ag in the kinetics of the recovery process in colored WO_3_ was investigated. This work provides a theoretical foundation for the design of hydrogen gasochromic materials with enhanced molecular oxygen activation ability to advance the recovery efficiency of the hydrogen gasochromic phenomenon.

## 2. Results and Discussion

### 2.1. The Optimization of Metal Promoters for Pt/WO_3_

**Figure 1:**
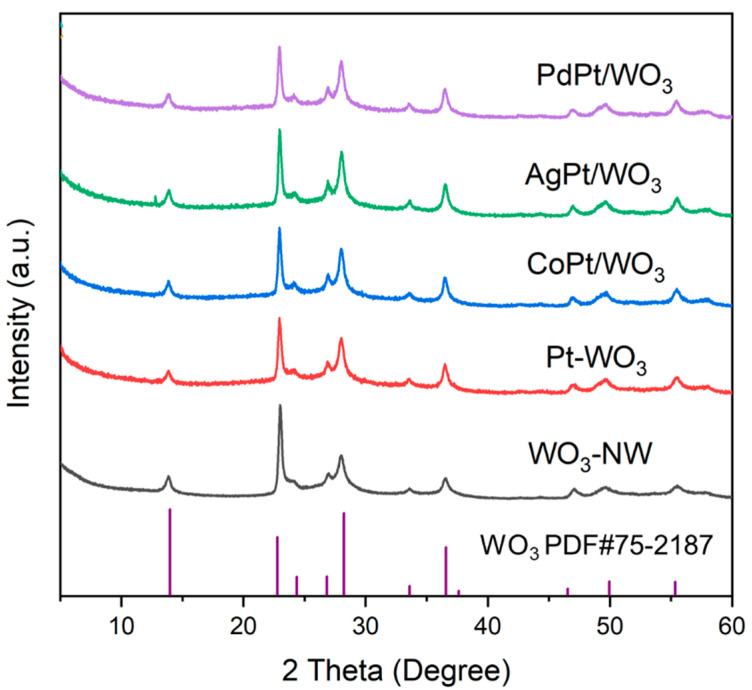
The XRD patterns of the as-prepared MPt/WO_3_ materials.

Figure 1 is the XRD patterns of the as-synthesized WO_3_, Pt/WO_3_, and nanostructured MPt/WO_3_ samples. The XRD pattern of WO_3_ was indexed as hexagonal phase (PDF # 75-2187), and the diffraction peaks at 14.0°, 22.8°, 28.2°, and 36.6° are indexed to the (100), (001), (200), and (201) planes of WO_3_. After the introduction of Pt and metal promoters, no diffraction peaks of Pt and metal promoters are visible in the XRD patterns of as-prepared samples, and the location of diffraction peaks is not shifted compared with WO_3_. Therefore, the reduction process has little influence on the crystal structure of WO_3_, and the introduced metal nanoparticles are highly dispersed on WO_3_, not reaching the detection limit of XRD.

**Figure 2:**
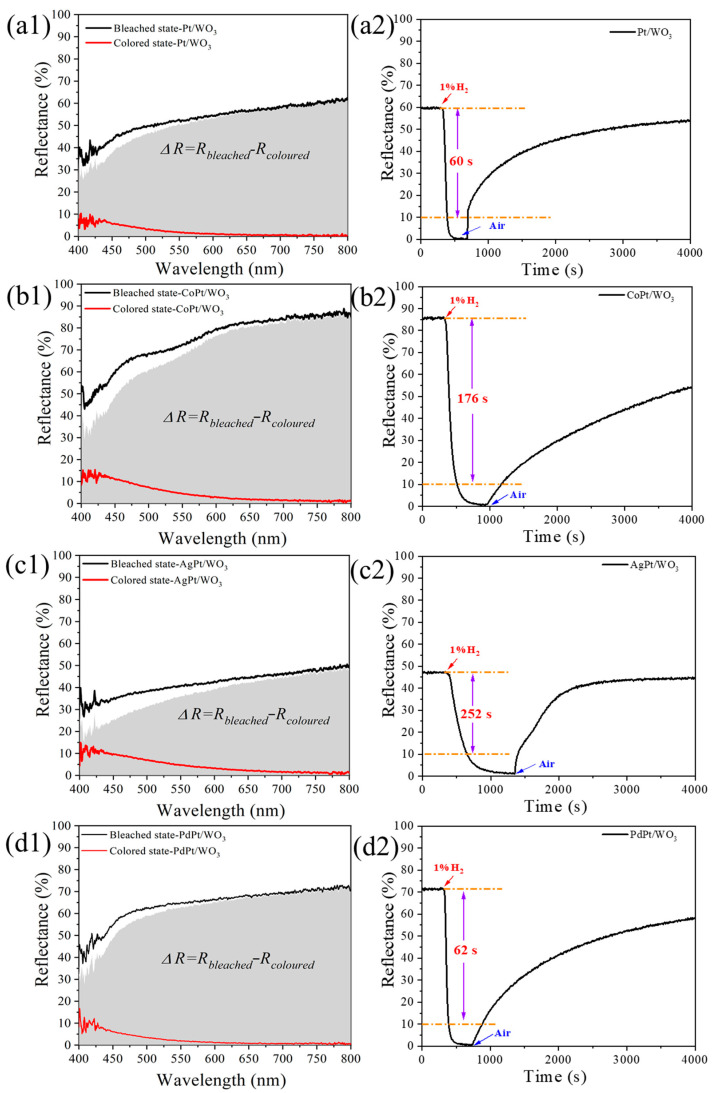
Reflectance spectra of (**a1**) Pt/WO_3_, (**b1**) CoPt/WO_3_, (**c1**) AgPt/WO_3_, and (**d1**) PdPt/WO_3_ in the bleached state and colored state, and the dependence of reflectance on time in coloring and bleaching for (**a2**) Pt/WO_3_, (**b2**) CoPt/WO_3_, (**c2**) AgPt/WO_3_, and (**d2**) PdPt/WO_3_ at the wavelength of 750 nm. The orange line represents the corresponding reflectance change with respect to the coloring time.

To quantitate the visible color change of WO_3_ samples during the coloring and bleaching process, the hydrogen gasochromic performance of MPt/WO_3_ powder samples was evaluated by monitoring the change in reflectance spectra upon exposing to 1% H_2_ and air sequentially. The spectra in Figure 2(a1–c1) represent the samples in a bleached or colored state before and after exposing to 1% H_2_-Ar gas. The gray area represents the variation in reflectance spectra in the range from 400 to 800 nm. The initial reflectance spectra of the CoPt/WO_3_ and PdPt/WO_3_ samples are similar to that of Pt/WO_3_, but the intensity of the initial spectrum for AgPt/WO_3_ was obviously decreased, indicating that the initial color of the sample became darker after introducing Ag. The resembled spectra for samples at colored state indicate that the four samples could finally reach similar dark blue states after contact with 1% H_2_.

To further evaluate the gasochromic performance disparities of samples during the coloring and bleaching stages, the alteration of spectral intensity at 750 nm was monitored by sequentially exposing samples to hydrogen and air. After exposing to 1% H_2_, the initial reflectance of all samples at 750 nm exhibited a dramatic reduction to nearly zero, indicating that the color of the WO_3_ sample had turned to the final dark-blue state. The variation in reflectance between the initial and final reflectance at 750 nm in the coloring stage was recorded as ΔR. In addition, the kinetics of the coloring and bleaching process of Pt/WO_3_ also present a distinct disparity after introducing the different metal promoters. The PdPt/WO_3_ exhibits a similar coloring time to that of Pt/WO_3_, but the coloring times of CoPt/WO_3_ and AgPt/WO_3_ samples were extended to 176 s and 252 s, respectively. After switching the atmosphere to air, the reflectance of all samples increases gradually, indicating that the colored samples begin to recover in air. Among the as-synthesized samples, only the AgPt/WO_3_ sample could recover to the initial bleached state within 12 min, while other samples could only partially recover even after being exposed in air for more than 50 min. In addition, it is worth noting that the bleaching in the initial recovery stage is relatively fast, and then gradually decreases over time for all samples.

**Figure 3:**
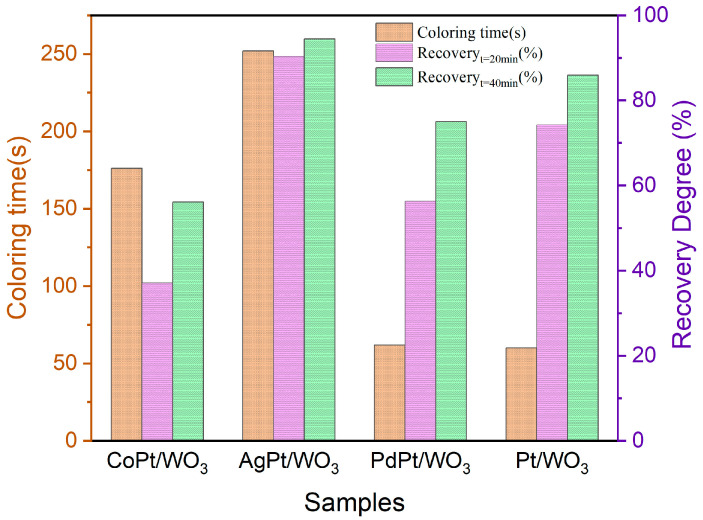
Hydrogen gasochromic performance comparison of Pt and as-prepared MPt/WO_3_ materials.

To further compare the color change and recovery performances of the four samples, statistics data on the coloring time and the recovery degree after recovering in air for 20 min and 40 min for Pt/WO_3_ and MPt/WO_3_ samples are presented in Figure 3. The recovery degree is defined as the ratio of recovered ΔRt within specific time frame (20 or 40 min) to the ΔR in the coloring stage. As shown in Figure 2(a2–c2), the introduction of Co suppresses both the coloration and recovery performance of Pt/WO_3_. In contrast, the addition of Pd has minimal impact on the gasochromic properties of Pt/WO_3_. After introducing Ag into the Pt/WO_3_ sample, the colorating time was extended. However, the sample was able to revert to the initial bleached state within 12 min, resulting in a significant improvement in the recovery degree. To obtain a more in-depth understanding of the role of Ag in promoting the recovery process, Ag was chosen as the target secondary metal for subsequent research.

### 2.2. Characterization of Pt/WO_3_ Materials with Ag Promoter

**Figure 4:**
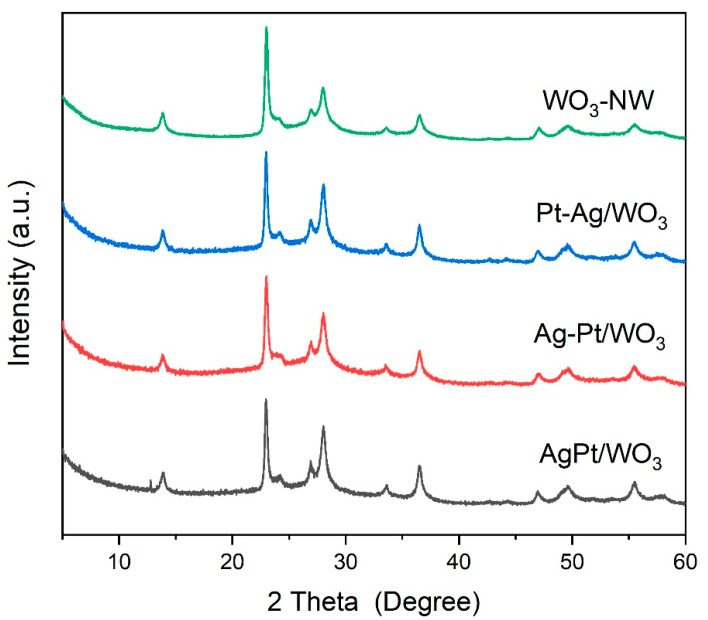
XRD patterns of Pt/WO_3_ materials with Ag promoter.

To investigate the promotional effect of Ag on the recovery process, metallic Pt and Ag were supported on WO_3_ nanowires in sequence. The sample with Pt introduced prior to Ag was designated as Pt-Ag/WO_3_, while the sample with Ag introduced prior to Pt was designated as Ag-Pt/WO_3_, and the sample with Ag introduced with Pt simultaneously was designated as AgPt/WO_3_. The phase and structure of different PtAg/WO_3_ samples were confirmed by XRD analysis (Figure 4). The XRD patterns indicated that no new peaks emerged, and the peak positions remained unchanged after the stepwise support process, suggesting that the step-deposition process did not affect the crystalline phase or structural integrity of WO_3_. Additionally, no diffraction peaks associated with Ag or Pt species were detected, which can be ascribed to the high dispersion of Ag and Pt active sites, and the size of these metal particles is below the detection limit of XRD.

**Figure 5:**
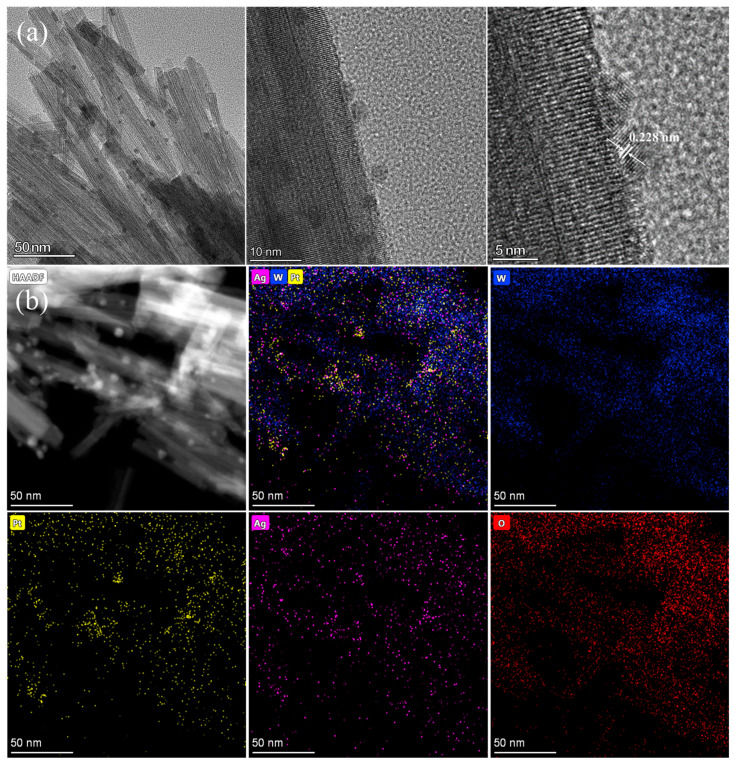
(**a**) (HR)-TEM images of AgPt/WO_3_; (**b**) STEM and mapping images of AgPt/WO_3_.

To further investigate the microstructure of the Pt/WO_3_ materials with the Ag promoter, transmission electron microscopy (STEM) was employed to investigate the influence of silver introduction sequences on the size and distribution of metallic nanoparticles in AgPt/WO_3_, Pt-Ag/WO_3_, and Ag-Pt/WO_3_ (Figure 5 and Figures S1–S3). Figure 5a shows that the AgPt/WO_3_ sample preserves the original nanowire morphology, further confirming that the deposition process of Ag and Pt has minimal impact on the morphology of WO_3_ nanowires. The observed particles are the highly dispersed metal nanoparticles on WO_3_, with an average particle about 3 nm. The well-resolved lattice fringes with an inter-planar spacing of 0.228 nm were calculated from the nanoparticles, verifying the good crystalline order of PtAg nanoparticles. The inter-planar spacing of AgPt lattice fringes is larger than that of the (111) crystal plane of metallic Pt (d_Pt (111)_ = 0.225 nm), and smaller than that of the (111) crystal plane of metallic Ag (d_Ag (111)_ = 0.235 nm), which is consistent with the alloy feature. In addition, high-angle annular dark-field scanning-transmission electron microscopy (HAADF-STEM) mappings were provided to study the composition distribution of the synthesized AgPt/WO_3_ sample (Figure 5b). Both Pt and Ag atoms are homogeneously distributed throughout the AgPt sample, which further demonstrates the alloy nature of Ag and Pt in the AgPt/WO_3_ product. Figure S2 shows the HAADF-STEM images and elemental mapping profiles of the Pt-Ag/WO_3_ sample. The small metal bright spots suggested that the Pt and Ag active sites are homogeneously dispersed on WO_3_. The elemental mapping profiles also verified the existence and uniform distribution of Pt and Ag elements. However, altering the support sequence to Ag followed by Pt influences the size and distribution of metal active sites. The HAADF dark-field image of the Ag-Pt/WO_3_ sample exhibits distinct metal particles with an average particle size of approximately 5 nm (Figure S3). Elemental mapping of Ag-Pt/WO_3_ demonstrates that the spatial distribution of Ag closely resembles that of Pt, implying that the subsequently introduced Pt is preferentially deposited on or adjacent to the pre-existing Ag sites, resulting in the formation of relatively large particles. However, the large AgPt active sites with Pt-surface-rich heterogeneous nanostructures not only reduce the dispersion degree of Pt and Ag, but also decrease the surface exposure ratio of Ag on the outer surface of nanoparticles.

**Figure 6:**
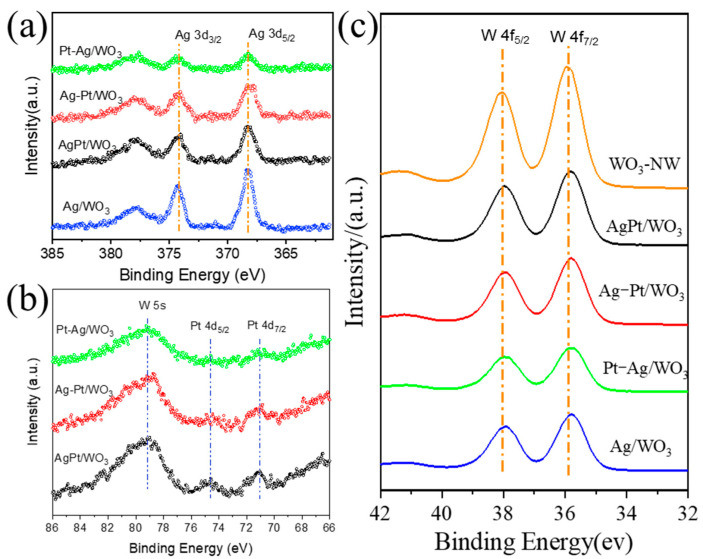
(**a**) Ag 3d XPS spectra, (**b**) Pt 4d XPS spectra, and (**c**) W 4f XPS spectra of WO_3_, Pt/WO_3_, Ag/WO_3_, and different PtAg/WO_3_ samples.

To investigate the valence states of elements and the interactions among different components, X-ray photoelectron spectroscopy (XPS) analyses were performed on WO_3_, Pt/WO_3_, Ag/WO_3_, and PtAg/WO_3_ samples, as presented in Figure 6. The high-resolution Ag 3d XPS spectra of the samples are presented in Figure 6a. The peaks located at 368.3 eV and 374.3 eV are assigned to metallic Ag^0^, corresponding to Ag 3d_5_/_2_ and Ag 3d_3_/_2_, respectively [24]. The variation in full width at half maximum (FWHM) reflects differences in the degree of silver reduction among the samples [25]. The Ag 3d XPS spectra of Ag-Pt/WO_3_ are similar to those of AgPt/WO_3_. In comparison with Ag/WO_3_, the higher FWHM of the Ag 3d_5/2_ peak in Pt-Ag/WO_3_ is primarily attributed to a lower extent of silver reduction [26]. In the Pt 4f spectra of the PtAg/WO_3_ samples with a different support sequence, the peaks at 71.2 eV and 74.6 eV are attributed to the Pt 4f_7_/_2_ and Pt 4f_5_/_2_ of metallic Pt^0^. Due to the low loading amount and high dispersion degree of Pt, the peak intensities are relatively weak. Compared with the PtAg/WO_3_ and Ag-Pt/WO_3_ sample, the Pt 4f XPS signal in Pt-Ag/WO_3_ was submerged in the background, implying that the sequential deposition of Ag may partially cover Pt active sites, thereby influencing the exposure and accessibility of Pt during the gasochromic process. The high-resolution W 4f XPS spectra display two peaks at binding energies of 35.9 eV and 38.1 eV, which are characteristic of W^6+^ species. After the support of Pt and Ag, the binding energy of the W^6+^ 4f_7/2_ peak shifted toward a lower binding energy. Notably, the shift extent has a close relationship with the proximity of Ag with the WO_3_ surface. The Ag/WO_3_ sample exhibits lower binding energy in the W 4f spectra compared with WO_3_, indicating that electron transfer occurs from Ag to the W species through the contact interface.

### 2.3. Hydrgen Gasochromic Properties of Pt/WO_3_ Materials with Ag Promoter

**Figure 7:**
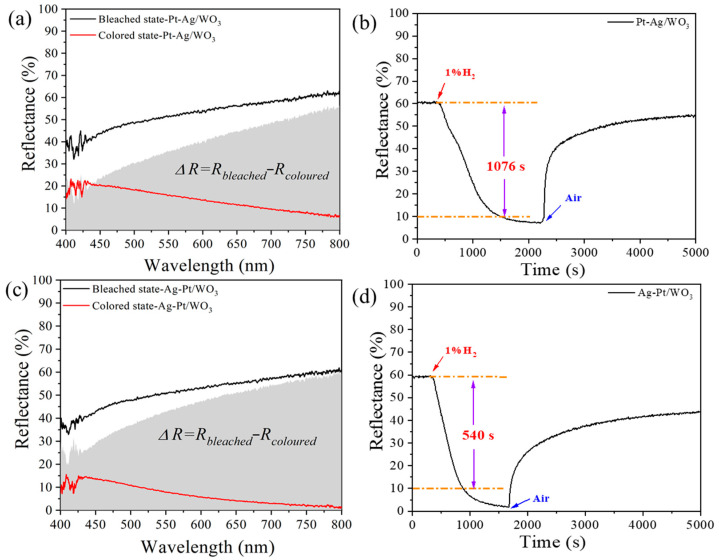
(**a**,**c**) are the reflectance spectra of the Pt-Ag/WO_3_ and Ag-Pt/WO_3_ samples; (**b**,**d**) are the dependence of reflectance on time in coloring and bleaching for the Pt-Ag/WO_3_ and Ag-Pt/WO_3_ samples. The orange line represents the corresponding reflectance change with respect to the coloring time.

The hydrogen gasochromic coloring and bleaching performances of the Pt-Ag/WO_3_ and Ag-Pt/WO_3_ samples were evaluated to reveal the effect of Ag on the coloring and bleaching process (Figure 7). As shown in Figure 7a,b, the initial reflectance spectrum of Pt-Ag/WO_3_ is similar to that of Pt/WO_3_, and exhibits obvious changes upon exposure to 1% H_2_. However, the coloration performance of Pt-Ag/WO_3_ is suppressed, which is due to the Ag deposited after Pt reduces the surface exposure and accessibility of Pt active sites, as confirmed by XPS spectra, thereby inhibiting the hydrogen dissociation and spillover process that are critical for the coloring process. In contrast, the bleaching process is significantly enhanced by the metallic Ag promoter. Upon exposure to air, the Pt-Ag/WO_3_ sample reverts to 50% of its recovery reflectance within 60 s, and the bleaching time of the Pt-Ag/WO_3_ sample was shortened to 488 s at room temperature, which is superior to that of Pt/WO_3_ and other previously reported crystalline materials [11,17].

Figure 7c,d illustrate the coloring and bleaching properties of the Ag-Pt/WO_3_ sample. The coloration performance of the Ag-Pt/WO_3_ sample is improved compared to that of the Pt-Ag/WO_3_ sample due to Pt-surface rich heterogeneous nanostructures. Therefore, the recovery behavior and response time of the Ag-Pt/WO_3_ sample are similar to those of the Pt/WO_3_ sample.

The statistical data for the coloration and bleaching process of the Pt/WO_3_ and PtAg/WO_3_ samples are presented in Table 1. Based on the aforementioned results and Table 1, the gasochromic and recovery properties exhibit a close relationship with the characteristics of the noble metal sites. In the case of the Pt/WO_3_ material, the highly dispersed Pt nanoparticles can efficiently dissociate hydrogen, and these dissociated hydrogen atoms can spill over to WO_3_ and react with the oxygen ions at the c-axis of WO_3_ nanowires, resulting in the formation of local water molecules [15]. Based on our previous research, the WO_3_ nanowires are favored for exposing the accessible oxygen at the c-axis and improving the gasochromic performance [17]. As for the recovery stage, the high initial bleaching rate indicates that the Pt nanoparticles could realize the rapid dissociation of oxygen, and the dissociated hydrogen in the WO_3_ structure tends to react with the dissociated oxygen. However, the gradual decrease in bleaching rate implies that the spillover of dissociated oxygen limits the following recovery process. Regarding the Ag-Pt/WO_3_ sample, the growing Pt was adhered to or covered the initially supported Ag particles, resulting in the formation of large metallic particles with a Pt-rich surface. These large particles with heterogeneous nanostructures could reduce the hydrogen dissociation efficiency, and extend the hydrogen spillover pathway, both of which impeded the coloring stage. The initial recovery process was also affected by this factor, but the subsequent bleaching behavior is similar to that of the Pt/WO_3_ sample.

For the AgPt/WO_3_ samples, the co-deposition of Ag and Pt results in the formation of metallic Ag-Pt nanoparticles. As a result, the gasochromic performance is influenced by the partial substitution of Ag with Pt on the surface, which reduces the availability of accessible Pt. In contrast, the uniform distribution of Ag can promote the activation of oxygen and assist the adjacent Pt species, resulting in improved recoverability. In the Pt-Ag/WO_3_ samples, although the Ag was deposited after Pt, the formed tiny Ag-Pt nanoparticles exhibited a high degree of dispersion. Consequently, the coloring rate was significantly affected because of partially inaccessible Pt inhibiting the hydrogen dissociation efficiency. During the recovery process, the highly dispersed Ag sites could effectively facilitate the oxygen dissociation [21,27], accelerate the recovery kinetics, and maintain the high bleaching rate over an extended period. However, the recovery rate decelerated in the final stage of recovery, indicating that the oxygen spillover in bulk becomes the main reason for the sluggish recovery of WO_3_ in this stage [9].

### 2.4. The Mechanism of Ag in Promoting Recovery

According to the local water molecule model [15], the initial step in the bleaching process of hydrogen gasochromic materials in the colored state involves the activation of O_2_ by surface Pt nanoparticles, leading to the formation of activated oxygen species. In the subsequent step, the activated oxygen diffuses to the WO_3_ surface, accepts electrons from the conduction band, and transforms into oxygen ions. This process results in an increase in surface oxygen concentration and a decline in hydrogen concentration, thereby promoting the decomposition of adjacent water molecules. The liberated oxygen atoms subsequently migrate into the lattice and occupy intrinsic oxygen vacancies, capturing electrons to reconstruct W-O bonds.

**Figure 8:**
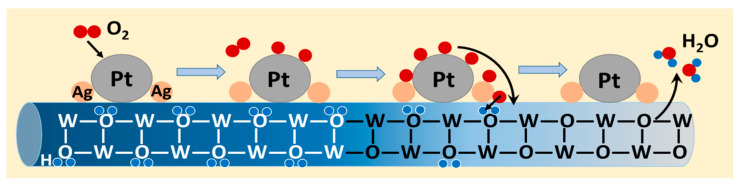
Schematic model of hydrogen gasochromic recovery for Ag-decorated Pt/WO_3_ NWs when exposed to O_2_.

The silver promoter possesses superior O_2_-activation capabilities, as Ag can optimize the adsorption of O_2_, and achieve efficient activation and dissociation of O_2_ molecules on WO_3_ under mild conditions, forming reactive oxygen species [23,28,29]. Therefore, those highly dispersed Ag nanoparticles can generate abundant reactive oxygen species (Figure 8), which will favor the spillover process and the efficient recovery of WO_3_ [22].

## 3. Materials and Methods

### 3.1. Materials

All reagents used in the experiments were commercial-grade and employed without further purification. Sodium tungstate, hydrochloric acid, hexachloroplatinic acid hexahydrate, and cobalt(II) nitrate hexahydrate were supplied by Sinopharm Chemical Reagent Co., Ltd. (Shanghai, China). Silver nitrate, palladium chloride, and sodium borohydride were acquired from Shanghai Aladdin Biochemical Technology Co., Ltd. (Shanghai, China), while anhydrous ethanol was provided by Shanghai Titan Scientific Co., Ltd. (Shanghai, China).

### 3.2. Synthesis of Metallic MPt/WO_3_ with Promoters

The WO_3_ nanowires were synthesized according to our previous report [17]. The obtained sample was named WO_3_-NW.

The obtained WO_3_-NW (0.5 g) was dispersed in deionized water (50 mL), and stirred until forming a uniform suspension. Then, 13.5 mL aqueous solutions of hexachloroplatinic acid aqueous solution (1 g/L) and second metal source (cobalt(II) nitrate hexahydrate (0.91 g/L) as the Co source, silver nitrate (0.3 g/L) as the Ag source, and palladium chloride (0.31 g/L) as the Pd source) solutions were added dropwise under stirring. After that, 13 mL of the NaBH_4_ solution (2 g/L) was added dropwise to the suspension under stirring. The obtained product was separated by filtration, washed several times with anhydrous ethanol, and subsequently dried in an oven at 80 °C for 6 h. The Pt and second metal contents are 1 wt% and 0.5 wt%, respectively. The final products were denoted as Pt/WO_3_, CoPt/WO_3_, AgPt/WO_3_, and PdPt/WO_3_, respectively.

To investigate the mechanism of the optimal second metal, Pt and Ag were supported on WO_3_ in different sequences according to the aforementioned procedure. The obtained products were labeled as Ag-Pt/WO_3_ and Pt-Ag/WO_3_.

### 3.3. Characterization

**Figure 9:**
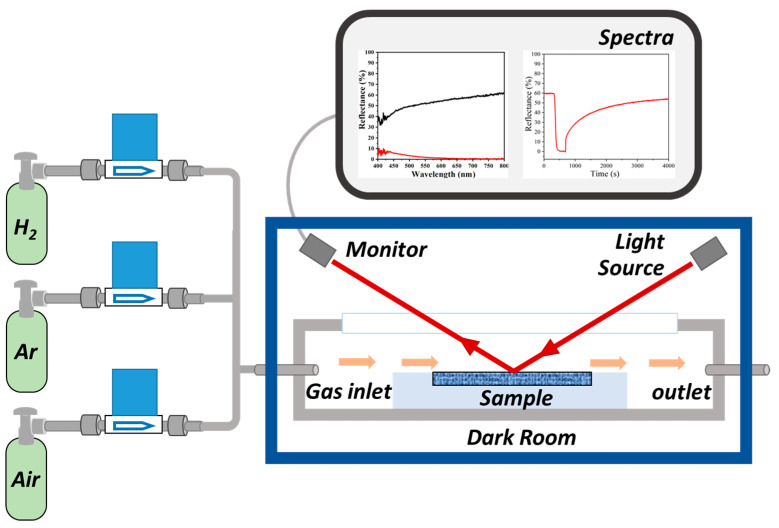
Scheme of gasochromic monitoring system for quantifying color change by visible light reflectance. The red line represents the light path in the monitoring system.

The crystal structure of the material was characterized using a Panalytical X’Pert PRO MPD X-ray diffractometer (Panalytical, Almelo, The Netherlands) equipped with Cu Kα radiation (λ = 0.15406 nm). The microstructure and the dispersion of the metal active sites were investigated by scanning transmission electron microscopy (STEM, TalosF200X, Thermo Scientific, Waltham, MA, USA). X-ray photoelectron spectroscopy (XPS) analyses were conducted on a Thermo Scientific ESCALAB 250Xi spectrometer (Thermo Fisher Scientific, Waltham, MA, USA). UV-vis spectroscopy (FLAME-UV-VIS system, Ocean Optics, Duiven, The Netherlands) was utilized to measure optical reflectance spectra and monitor the coloration/bleaching behavior in response to exposure in hydrogen and air. The dynamic color variation was assessed by tracking the changes in reflectance intensity at specific wavelengths before and after H_2_ introduction. Before the testing, the flow rate was set to 60 sccm, and the concentration was modulated to 1% H_2_/Ar by mass-flow controllers (MFCs) using 5% H_2_/Ar and pure Ar gases. The real-time optical signal change of the samples was determined by UV-vis spectroscopy, which is shown in Figure 9.

## 4. Conclusions

In summary, crystalline WO_3_ nanowires decorated with PtAg nanoparticles were synthesized by the chemical reduction deposition method, which exhibited improved recovery performance compared to Pt/WO_3_ in the H_2_ gasochromic process. The AgPt/WO_3_ gasochromic material exhibits a high response (ΔR = 45.2% in 1% H_2_) and high recovery degree (94%) at room temperature. The assistance mechanism of Ag is closely correlated with its high dispersion and the proximity with Pt. The catalytic effect of Ag in O_2_ dissociation is crucial for enhancing the oxygen spillover in bulk and recovery kinetics of WO_3_. Additionally, the large exposure ratio of oxygen on the c-axis shortens the diffusion distance of oxygen spillover. The proximity of highly distributed Ag and Pt nanoparticles could exhibit an enhanced synergistic effect and promote the constant rapid activation of oxygen through spillover during the recovery process, and the recovery time could be reduced to one fifth that of Pt/WO_3_. This work provides a new prospective to develop H_2_ sensors with high sensitivity to both hydrogen and oxygen, to ensure the practical and safe usage of H_2_.

## Figures and Tables

**Table 1:** Gasochromic properties of Pt/WO_3_, AgPt/WO_3_, Pt-Ag/WO_3_, and Ag-Pt/WO_3_ samples.

Sample	ΔR ^1^ [%]	t_C_ ^2^ [s]	r_B_ ^3^ [%·s^−1^]	t_R50%_ ^4^ [s]	t_R60%_ ^5^ [s]	t_R70%_ ^6^ [s]	t_R80%_ ^7^ [s]	Recovery Degree ^8^
Pt-Ag/WO_3_	52.9	1076	0.783	52	104	220	488	89.5%
Ag-Pt/WO_3_	57.5	540	0.443	212	368	636	1020	72.5%
AgPt/WO_3_	45.2	252	0.393	308	388	492	636	94.0%
Pt/WO_3_	59.1	60	0.836	260	428	672	1056	90.1%

^1^ Changes in reflectance in coloring stage. ^2^ Coloring time. ^3^ Initial Bleaching rate, a percentage of transmittance per second (%/s) at initial recovery stage. ^4^ Bleaching time when recovering to 50% ΔRr (ΔRr represents changes in reflectance between colored state and final recovered bleached state). ^5^ Bleaching time when recovering to 60% ΔRr. ^6^ Bleaching time when recovering to 70% ΔRr. ^7^ Bleaching time when recovering to 80% ΔRr. ^8^ Ratio of final recovered ΔRr to initial ΔR in coloring stage.

## Data Availability

The data presented in this study are available on request from the corresponding author.

## Supplementary Materials

The following supporting information can be downloaded at: https://www.mdpi.com/article/10.3390/ijms27020833/s1.
