# Supplementary material for: Effect of Silver Promoter on the H2 Gasochromic Recovery Behavior of Pt-Decorated WO3 Nanowires
Source: Int J Mol Sci. 2026 Jan 14;27(2):833. doi: 10.3390/ijms27020833 (PMC12840972; doi:10.3390/ijms27020833)
Supplement: Supplementary file 1 [file ijms-27-00833-s001.zip › ijms-4053069-supplementary.pdf]

Supporting Information

Effect of silver promoter on the H_2 gasochromic recovery behavior of Pt-decorated WO_3 nanowires

Dandan Liu [1], Ziheng Geng [1], Aiyan Han [1], Rongjiao Che [1], Ping Yu [2], Huan Liu [2,*], Yunqi Liu [1,*]

[1] State Key Laboratory of Heavy Oil Processing, College of New Energy, College of Chemistry and Chemical Engineering, China University of Petroleum (East China), Qingdao, P. R. China

[2] State Key Laboratory of Chemical Safety, Sinopec Research Institute of Safety Engineering Co., Ltd., Qingdao, P. R. China

* Correspondence: liuyq-group@upc.edu.cn (Yunqi Liu), liuh.qday@sinopec.com (Huan Liu)

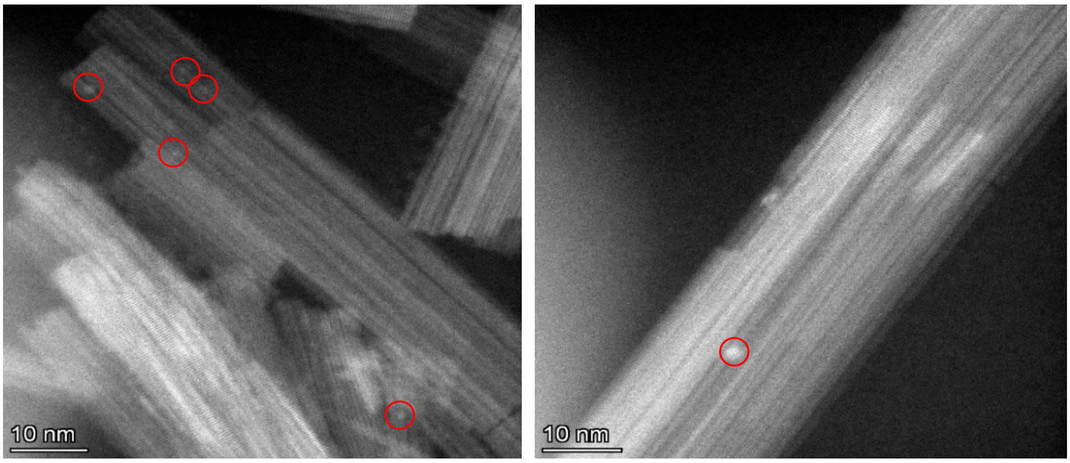

Figure S1. STEM images of Pt/WO_3.

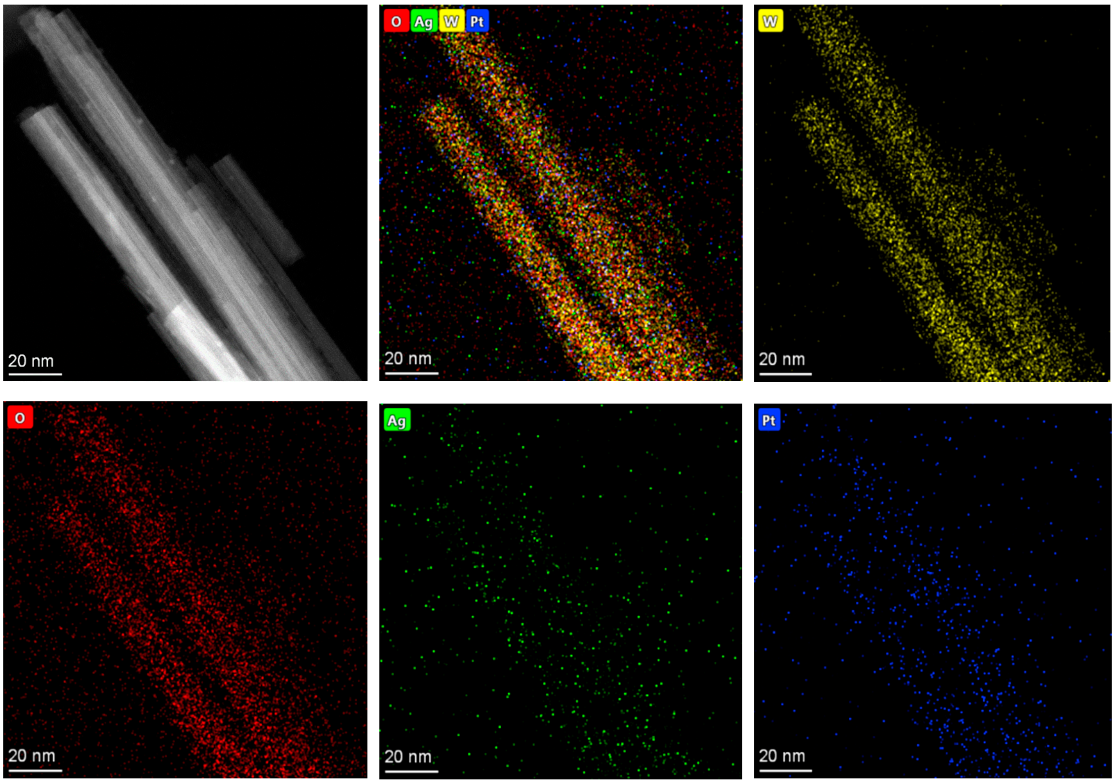

Figure S2. STEM and mapping images of Pt-Ag/WO_3.

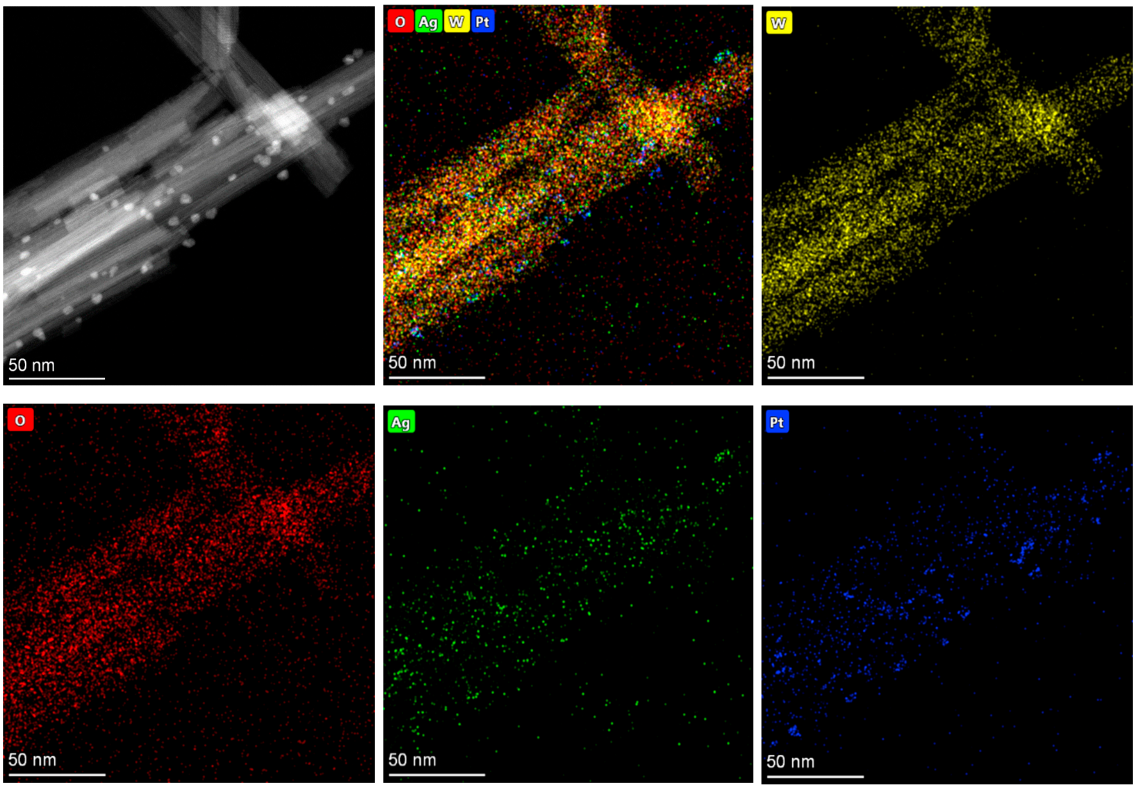

Figure S3. STEM and mapping images of Ag-Pt/WO_3 samples.
